# Influence of the solvent on the stability of bis(terpyridine) structures on graphite

**DOI:** 10.3762/bjnano.4.29

**Published:** 2013-04-22

**Authors:** Daniela Künzel, Axel Groß

**Affiliations:** 1 Institute of Theoretical Chemistry, Ulm University, D-89069 Ulm, Germany

**Keywords:** computer simulations, energy related, force-field calculations, nanomaterials, solvation

## Abstract

The effect of solvation on the adsorption of organic molecules on graphite at room temperature has been addressed with force-field molecular dynamics simulations. As a model system, the solvation of a bis(terpyridine) isomer in water and 1,2,4-trichlorobenzene was studied with an explicit solvation model. The inclusion of solvation has a noticeable effect on adsorption energies. Although the results of the various considered force fields differ quite significantly, they all agree that the adsorption of BTP from the TCB solvent is almost thermoneutral. The substrate simply acts as a template to allow a planar arrangement of the network, which is stabilized by the intermolecular interaction. Using an atomic thermodynamics approach, the order of the stability of various network structures as a function of the chemical potential is derived yielding a sequence in agreement with the experiment.

## Introduction

The controlled formation of structured surfaces by the formation of hydrogen-bonded organic networks is of technological interest for future applications such as molecular electronics, organic photovoltaics [1] or functionalized host–guest systems [2] that may be used in heterogeneous catalysis. As a model system for ordered organic adlayers, bis(terpyridines) (BTPs) have been studied intensively in recent years [2–10]. They are known to adsorb in a flat configuration on various surfaces and to form self-organized ordered surface structures. In previous publications, we were able to show that combined DFT and force-field simulations can help to explain experimental observations in the adsorption behavior of BTPs on graphite [11–12]. One example is the observation of blurred STM images of phthalocyanine molecules adsorbed as guest molecules in a BTP host network, which is due to the fact that rotations of the host molecules are hardly hindered by barriers [6,11].

Recently it was shown by scanning tunneling microscopy (STM) experiments that 3,3′-BTP exhibits a variety of adlayer structures at the interface between highly oriented pyrolytic graphite (HOPG) and the liquid as a function of the concentration in solution [6]. The resulting structures, i.e., one hexagonal, two closely related linear, and one densely packed linear structure, were ordered according to their packing density as a function of the concentration. Furthermore, it was found that the presence of the liquid has a decisive influence on the structure formation: whereas at the liquid/HOPG interface three closely related linear patterns and one hexagonal two-dimensional pattern were identified, at the gas/HOPG interface only one of the linear patterns and the hexagonal structure were found. The concentration dependence of the different surface structures was rationalized within a thermodynamic model [13]. However, in the calculations of the adsorption energies the solvent was entirely neglected, as is typically done in calculations addressing the adsorption of organic molecules [14], even if experimentally they are deposited from a solution.

Hence, we here address the adsorption of BTP on graphite in the presence of a liquid phase in order to assess the explicit influence of the solvent on the molecular adsorption at the solid/liquid interface. Note that the modeling of a liquid requires the determination of free energies instead of just total energies, which means that computationally expensive statistical averages have to be performed in order to evaluate free-energy differences. Although electronic structure calculations based on density functional theory can reproduce the properties of planar arrangements of aromatic molecules satisfactorily [15–18], the large size of the considered systems and the requirement to perform thermal averages make first-principles electronic-structure calculations computationally prohibitively expensive. Therefore we employed classical force fields as included in the Forcite module of the Accelrys’ Materials Studio package to describe the interaction between adsorbate, substrate and solvent. It is true that the force fields in this package tend to overestimate BTP adsorption energies on graphite [12]. Still, trends in the stability of BTP stuctures on graphite as a function of the environment should still be reproduced.

As a solvent, we have taken into account 1,2,4-trichlorobenzene (TCB), which was used in the experimental work [6]. Additionally, we have also considered water as a reference since many organic molecules are deposited from aqueous solutions. In this work, we show that the molecule–solvent interaction has an important influence on the stability range of the considered structures. Still, the order of the stability as a function of the chemical potential is not modified by the inclusion of the solvent effects. Because of the strong TCB–BTP interaction, the adsorption of a single BTP molecule on graphite out of a TCB solution is almost thermoneutral. Hence, it is the intermolecular interaction in the hydrogen-bonded networks on graphite that stabilizes the molecular layers; the surface just acts as a template to allow a planar arrangement of the hydrogen-bonded network.

## Computational details

**Figure 1:**
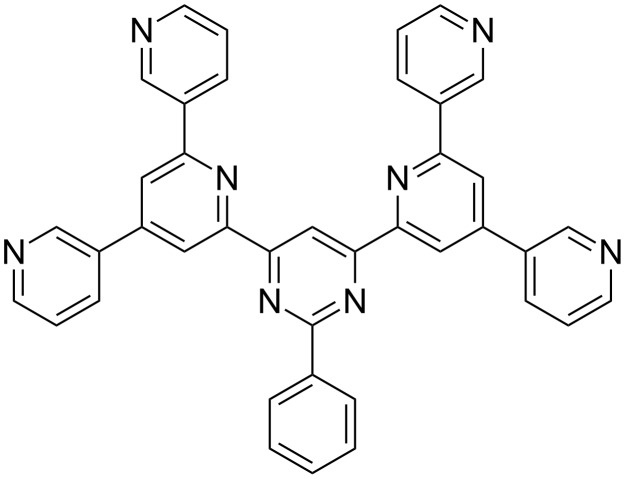
Structure of the 3,3′-BTP molecule.

In this study, force-field molecular dynamics are used in order to describe the adsorption properties of solvated BTP molecules on graphite. The structure of 3,3′-BTP, which is known for its high versatility in surface structures is shown in Figure 1. There are of course force fields that reproduce structural properties of water quite satisfactorily [19–20]. However, here we need general-purpose force fields that are able to describe different solvents, solvent–molecule and molecule–surface interactions equally well. Hence, we use the Universal (UFF) [21], Compass (condensed-phase optimized molecular potentials for atomistic simulation studies) [22], Dreiding [23] and Consistent Valence (CVFF) [24] force fields included in the Forcite module of the Accelrys’ Materials Studio package.

The graphite surface is modeled by a five-layer graphite (0001) slab. Convergence criteria are chosen according to the ultrafine settings of the program. Partial charges of the atoms are assigned with the Gasteiger [25] and QEq [26] methods for UFF and Dreiding, whereas charging methods are already included in the CVFF and Compass force fields.

As mentioned in the introduction, the theoretical treatment of liquids requires a consideration of the free energies and free-energy differences. Typically, free energy differences are determined by performing constrained MD simulations using either umbrella sampling schemes [27–28], free-energy perturbation methods [29] or some other appropriate thermodynamic integration scheme, such as the recently developed enveloping distribution sampling (EDS) method [30].

**Figure 2:**
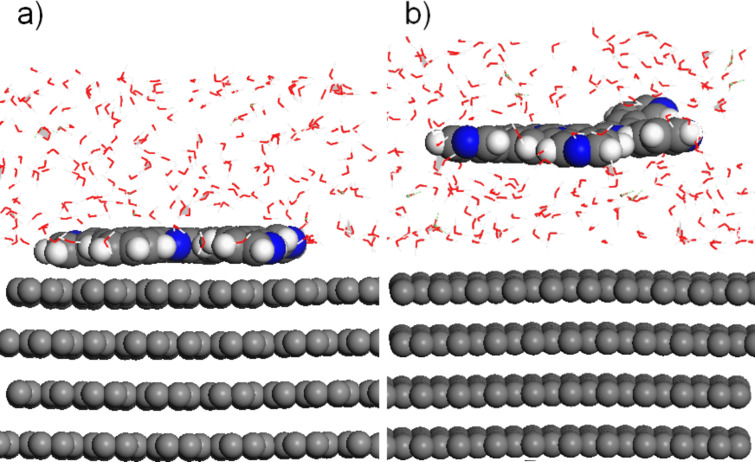
Structural models used to derive the free enthalpy of adsorption of a dissolved molecule: (a) adsorbed molecule at the substrate/solvent interface and (b) dissolved molecule above the substrate/solvent interface.

However, using one of these schemes often requires a series of molecular dynamics simulations. In order to derive the adsorption energy of the BTP molecules from solution at finite temperatures, we rather take advantage of the fact that BTP molecules on the surface and in solution replace approximately the same amount of solvent molecules. Hence, we determine the free enthalpy of adsorption 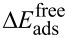 from the solvent according to the scheme illustrated in Figure 2, i.e., it is evaluated as the difference of the free enthalpy of the molecule adsorbed at the substrate/solvent interface minus the free enthalpy of the molecule dissolved above the substrate/solvent interface:

[1]
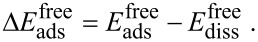


We also determine free enthalpies instead of free energies, in order to remain consistent with our previous thermodynamics calculations that we want to improve by using the solvent model. The free enthalpies are derived as the energy average through the molecular dynamics simulations, which were performed within the NPT ensemble at 298 K (Nosé thermostat) and at 0.0001 GPa (Berendsen barostat) after initial geometry optimization steps of the randomly chosen starting configuration according to [31]. The first 50 ps of the simulations with a time step of 1 fs were considered as the equilibration time; all averages were performed by using the subsequent 100–150 ps of simulation time.

The free enthalpies of adsorption in the presence of the solvent will be contrasted with the adsorption energies at the solid/gas interface, which were calculated as usual according to [32]

[2]
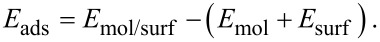


where *E*_mol/surf_ is the total energy of the molecule/surface system, and *E*_mol_ and *E*_surf_ are the total energies of the isolated molecule and the surface alone, respectively.

In order to validate the reliability of the force fields used in this study, we have considered liquid densities and the solvation energies in water and TCB. In order to be consistent with our scheme to determine the free enthalpies of adsorption, we estimated the solvation energy *E*_solv_ from the free enthalpy of the dissolved molecule 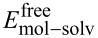, the free enthalpy of the solvent alone 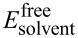, and the enthalpy of the isolated molecule *E*_mol_ according to

[3]
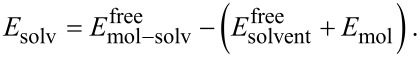


This procedure neglects effects due to the volume change of the solvent when the molecule is dissolved. However, due to the large number of solvent molecules included in the simulations, the influence of these effects should be negligible.

## Results and Discussion

### Validation step 1: liquid densities

As a first test case, the densities of liquid water and 1,2,4-trichlorobenzene (TCB) are considered, yielding an indication as to whether the intermolecular interactions within the solvent phase can be reproduced correctly by a force field. The calculated results are compared with the corresponding experimental values for water [33] and TCB [34].

**Figure 3:**
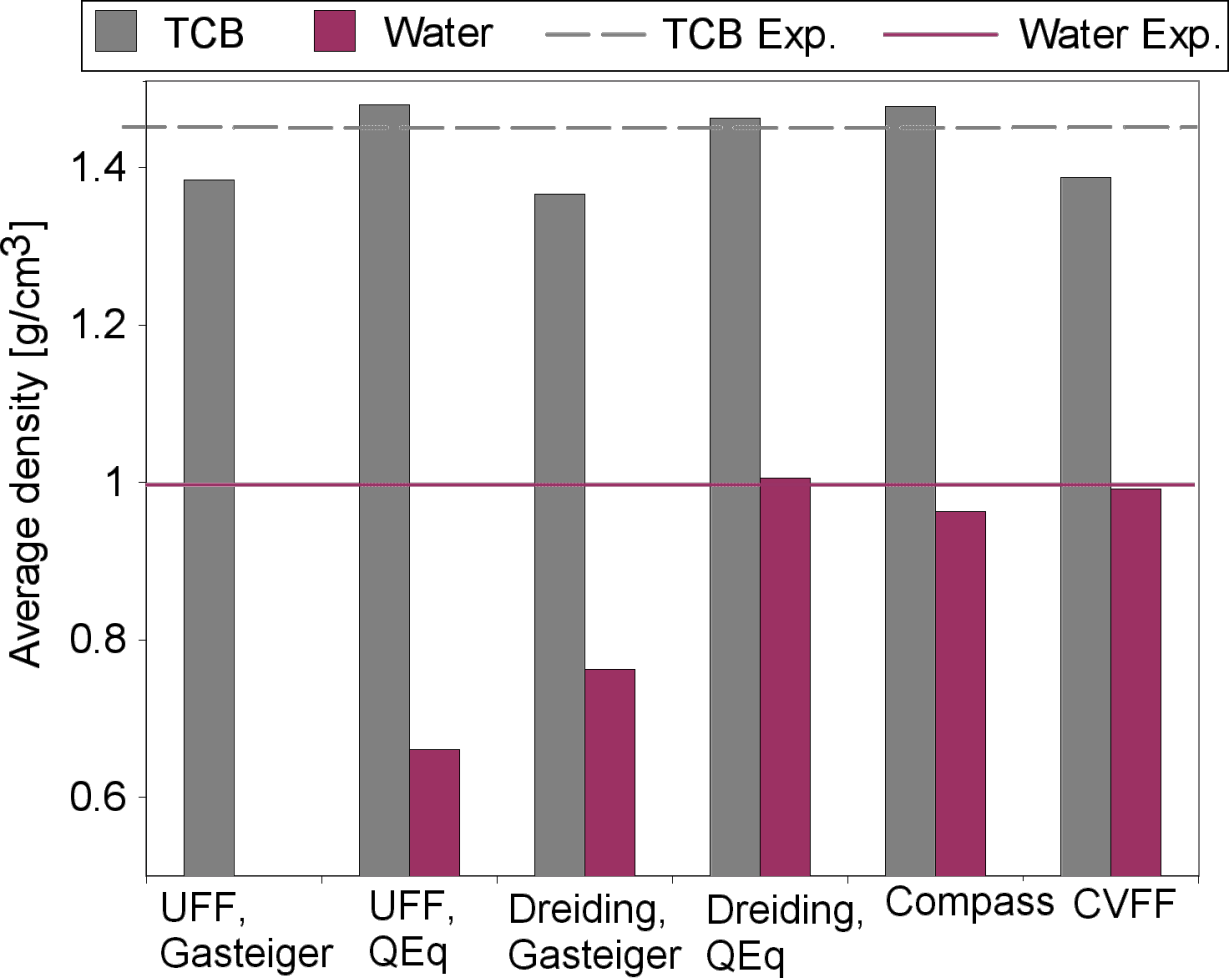
Force-field molecular dynamics densities of water and TCB at 298 K. Experimental values for water are taken from [33] and for TCB from [34].

For water, a strong variation between the different force-field results is observed (Figure 3). The average densities of the molecular dynamics trajectory range from 0.07 g/cm^3^ for UFF with Gasteiger charging, up to 1.01 g/cm^3^ for Dreiding with QEq charges. A value of 0.997 g/cm^3^ would have been expected [33]. With UFF, the deviation from the experiment is particularly high with both charging methods. Dreiding performs well with QEq charging, but not with Gasteiger charges. Compass and CVFF also show a deviation from experimental values of less than 5%. Further Compass calculations with varying system size could show that the solvent density does not change noticeably over a wide range of system sizes. Starting from a system of 30 water molecules, the density remains at 0.96 g/cm^3^. Using fewer water molecules leads to higher densities. But even when only three molecules are used, the density only increases by 8% to 1.04 g/cm^3^.

The energy difference between an isolated molecule and a molecule in the condensed phase, the cohesive energy, is also relatively independent of the system size. In systems of 10 to 700 water molecules, the cohesive energy remains at −365 to −369 meV. With smaller systems, the cohesive energy decreases: With five molecules representing liquid water, it drops to −404 meV. We also checked the influence of the runtime of the trajectories on the average values. Total runtimes of 150 ps are used in order to evaluate the influence of the length of equilibration phase and actual trajectory. For the larger systems, the extreme cases of 20 ps equilibration time and 130 ps runtime on the one hand, and of 100 ps equilibration and only 50 ps runtime on the other hand differ in average potential energy by only a few millielectronvolts. The standard deviation in the potential energy remains below 10 meV per molecule for cell sizes above ten molecules.

**Figure 4:**
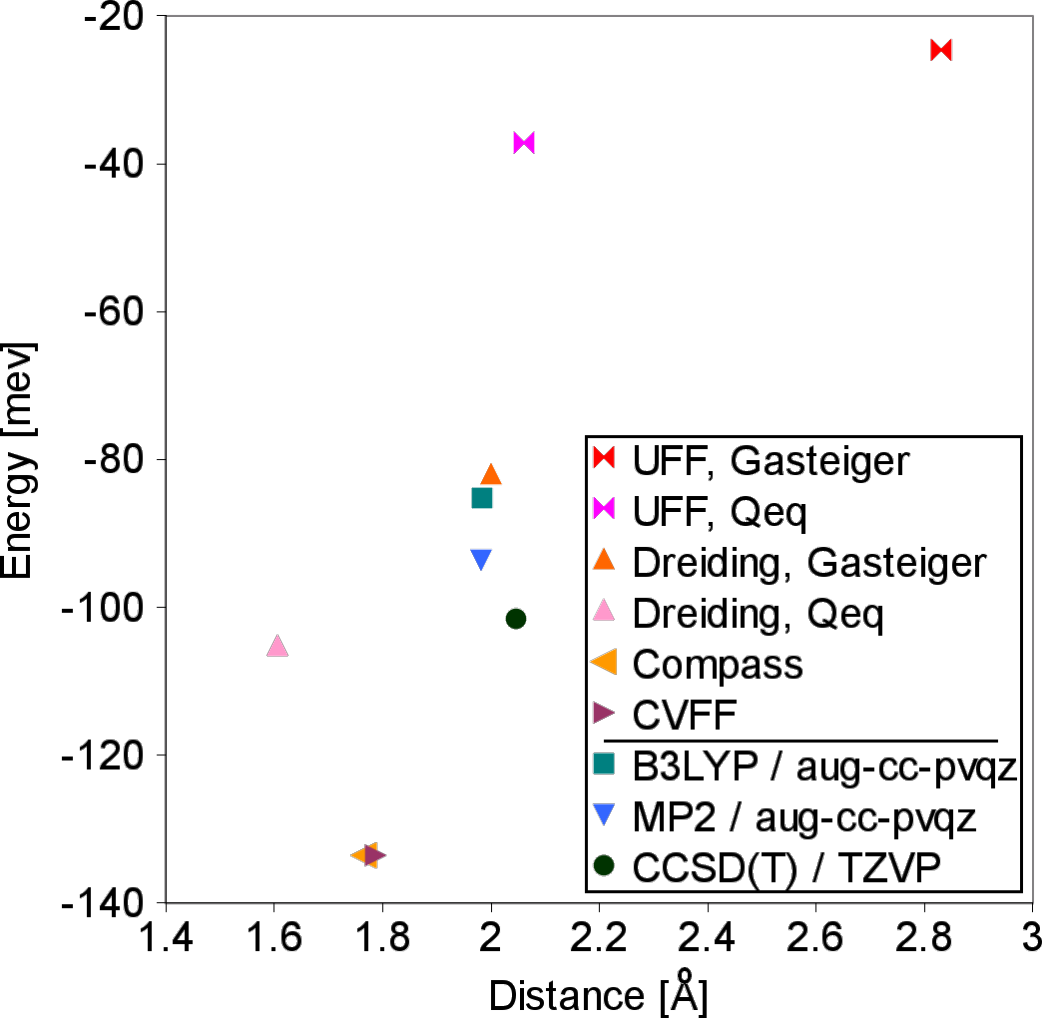
Equilibrium distance and interaction energy for water dimers, calculated with different force fields and with quantum chemical methods.

In order to understand the reason for the discrepancy between calculated water densities and the experimental value, we determined the equilibrium O–H distance and interaction energy for a water dimer with different force fields and additionally also with quantum chemical methods. The corresponding results are plotted in Figure 4. The considered quantum chemical methods agree with an equilibrium distance of 1.98 to 2.05 Å, with interaction energies ranging from −85 to −102 meV. Interestingly enough, Dreiding with Gasteiger charging reaches a very similar result of 2.00 Å and −82 meV, but still the Dreiding/Gasteiger density is much too low. Dreiding/QEq, Compass and CVFF have stronger hydrogen bonds of 105 to 137 meV. Although they yield water–water distances that are too small, their densities agree very well with the experimental result. With less than 40 meV, UFF greatly underestimates the hydrogen bonds, resulting in particularly low densities.

TCB on the other hand is more accurately described by force fields. The force field densities vary between 1.37 g/cm^3^ (Dreiding/Gasteiger) and 1.48 g/cm^3^ (UFF/QEq). The deviation from the experimental density of 1.45 g/cm^3^ [34] is less than 6% for all force fields.

In conclusion of this section, it is important to note that liquid water is only poorly reproduced by the force fields considered in this study due to problems with the reliable description of intermolecular hydrogen bonds and liquid densities. For TCB on the other hand, the force-field results are reasonably accurate, possibly because hydrogen bonds are less important in the TCB bonding.

### Validation step 2: solvation energies

**Figure 5:**
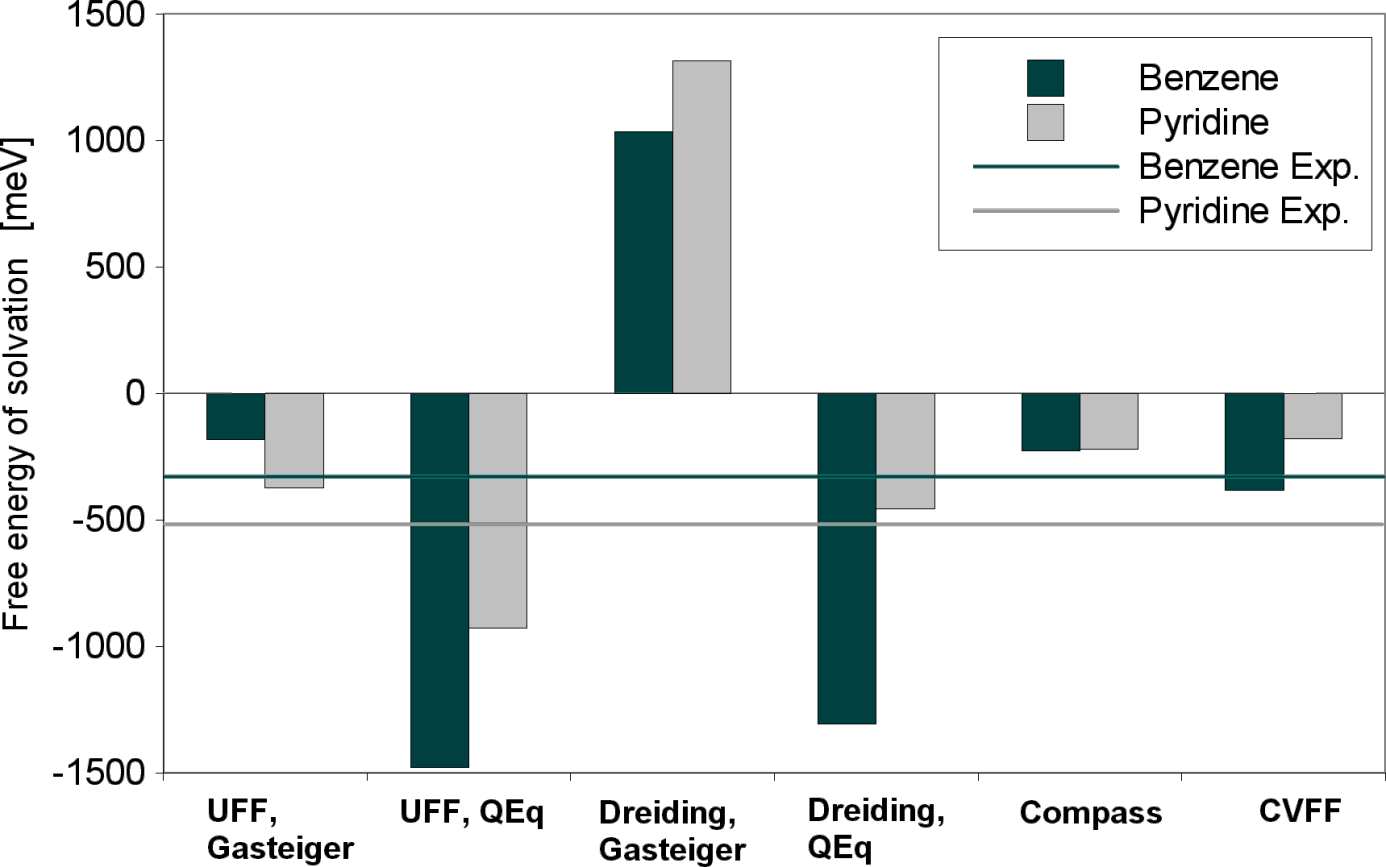
Force field molecular dynamics result for the free energy of solvation *E*_solv_ for pyridine and benzene in water. Experimental values taken from [35].

As a further validation, we addressed the interaction between solvent and dissolved organic molecule, which should be reproduced accurately for a meaningful description of the system. As test systems, we considered the solvation of pyridine and benzene as small-but-similar models for the larger BTP molecule, which consists of pyridine and benzene rings. The resulting solvation energies evaluated according to Equation 3 are collected in Figure 5 and compared with the experimental solvation energies of −517 meV for pyridine and −329 meV for benzene in water [35].

Quantitatively, most of the force field results do not agree very well with the experiment. Dreiding/QEq reproduces 88% of the pyridine solvation energy. CVFF describes the benzene solvation rather well: it overestimates the energy by only 16%. UFF/Gasteiger is correct in a qualitative sense, pyridine has a higher gain in solvation energy than benzene. This is not achieved by any of the other force fields. Still, UFF/Gasteiger underestimates the solvation energies.

Both UFF/QEq and Dreiding/QEq calculations fail for benzene solvation, they overestimate the solvation energy by a factor of 3 to 4. On the other hand, pyridine solvation energies are too small by a factor of 2 to 3 with CVFF and Compass. Dreiding/Gasteiger calculations result in positive solvation energies, whereas negative values would be expected.

These results are certainly not satisfactory in a quantitative way. Obviously the problem with the description of the intermolecular hydrogen bonds directly translates to inaccurate solvation energies in water. Still, most of the force fields are able to show that pyridine and benzene have small negative solvation energies in water, so the method might still be useful for a more qualitative analysis of the BTP adsorption process.

Another problem might be the rather crude model we use that neglects the changes of volume in the system. However, if the number of water molecules is large enough, this volume change becomes smaller than the natural fluctuations in the volume throughout the trajectory. For the benzene in water case, with 300 water molecules the volume changes by 6% when a benzene molecule is added to the system whereas the standard deviation amounts to 8% of the average volume for the benzene–water solvated system. With a further increase of the system size to 600 water molecules, the volume change amounts to less than 3%. Using more than 1200 water molecules brings about only small changes: For system sizes between 1200 and 2100 atoms, the volume change stays close to 1%, similar to the standard deviation.

**Figure 6:**
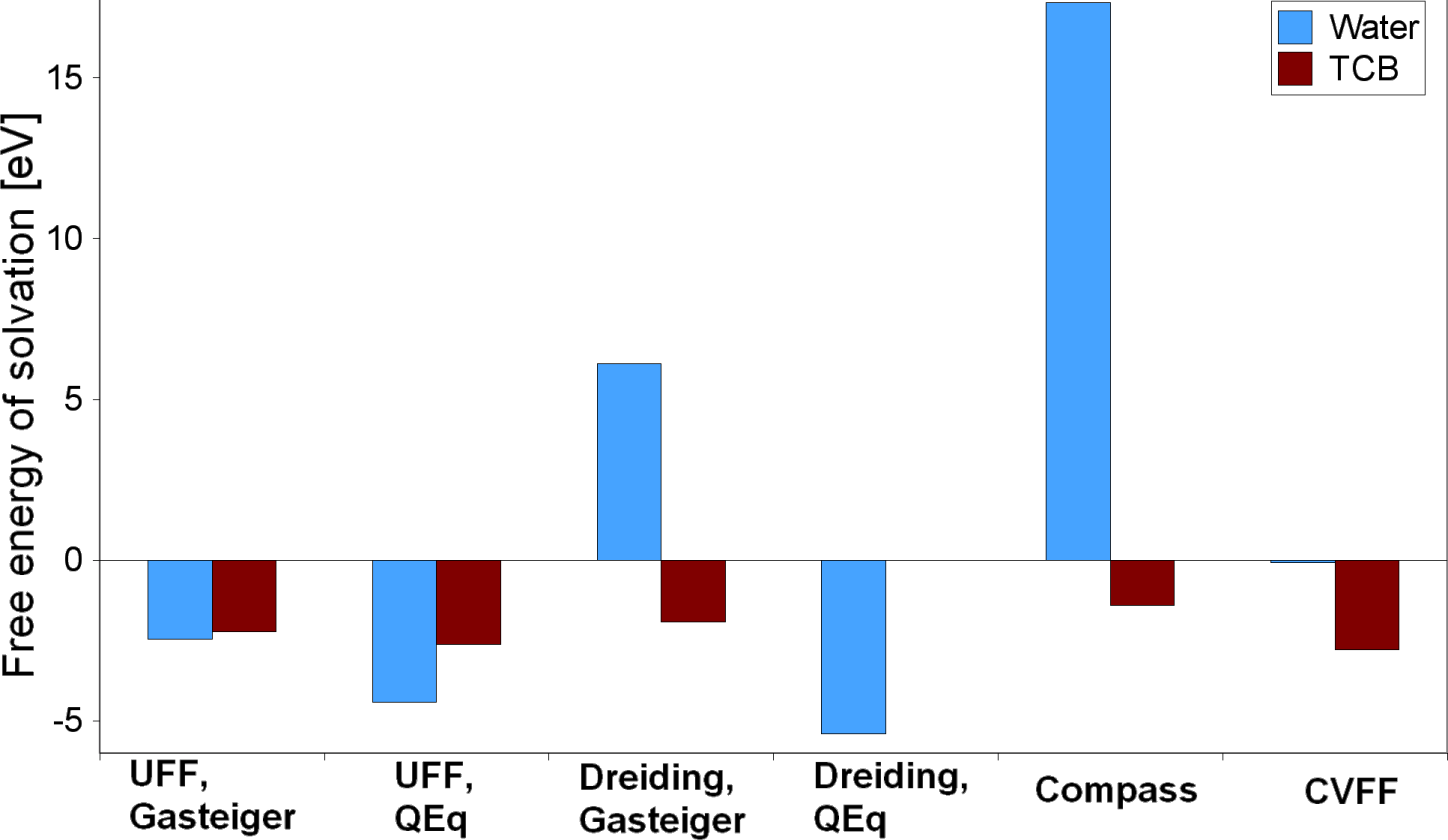
Force-field molecular dynamics result for the free energy of solvation *E*_solv_ for 3,3′-BTP in water and TCB.

Additionally, the BTP solvation process has been addressed. Note that due to the approximate nature of the determination of the solvation energy according to Equation 3 the results that are collected in Figure 6 and Table 1 can only be of a qualitative manner. However, to the best of our knowledge, there are no experimental values for the BTP solvation energies available. It is simply known that BTP molecules dissolve easily in TCB but are not soluble in water [4].

**Table 1:** Solvation (*E*_solv_) and adsorption (*E*_ads_) energies of 3,3′-BTP in eV calculated using different force fields.

	*E*_solv_		*E*_ads_		
	Water	TCB	Water	TCB	Vacuum

UFF, Gasteiger	−2.459	−2.225	−4.270	0.137	−4.560
UFF, QEq	−4.413	−2.625			
Dreiding, Gasteiger	6.112	−1.925	−2.696	−0.142	−3.941
Dreiding, QEq	−5.404	−0.016			
Compass	17.349	−1.406	−1.409	0.072	−4.027
CVFF	−0.068	−2.787	−3.569	−0.052	−7.312

Experiment				−0.340 [2]	−2.54 [12]

For the molecular dynamics simulations of the solvated BTP molecule, rather large unit cells containing 395 to 400 water molecules or 106 to 143 TCB molecules were used. This is a compromise between cells being large enough for minimal volume effects and being small enough for an efficient computational treatment.

The solvation energies of BTP in water and in TCB again vary strongly with the force field used. Not all force fields can reproduce the experimental findings at least in a qualitative way. Both UFF calculations and Dreiding/QEq result in a higher energy gain for the dissolution in water. With about 200 meV, the difference between solvation in water and TCB is only small for UFF/Gasteiger. It is more significant in the UFF/QEq and the Dreiding/QEq calculations with nearly 2 eV and more than 5 eV, respectively. Thus QEq charging is not used any more for the subsequent calculations.

Only with Compass, CVFF and Dreiding/Gasteiger, do the force field results show that it is energetically more favorable to dissolve the BTP molecule in TCB than in water. The Compass result is probably closest to the experimental observations. Here, the solvation in water is clearly not favorable, and the difference from the TCB solvation is more than 18 eV. Dreiding/Gasteiger show a similar trend, but with 8 eV, the energy difference is considerably smaller. In the CVFF calculation, the difference is only about 3 eV and the solvation in water is not decidedly unfavorable from an energetic point of view.

### Adsorption of a dissolved BTP molecule

**Figure 7:**
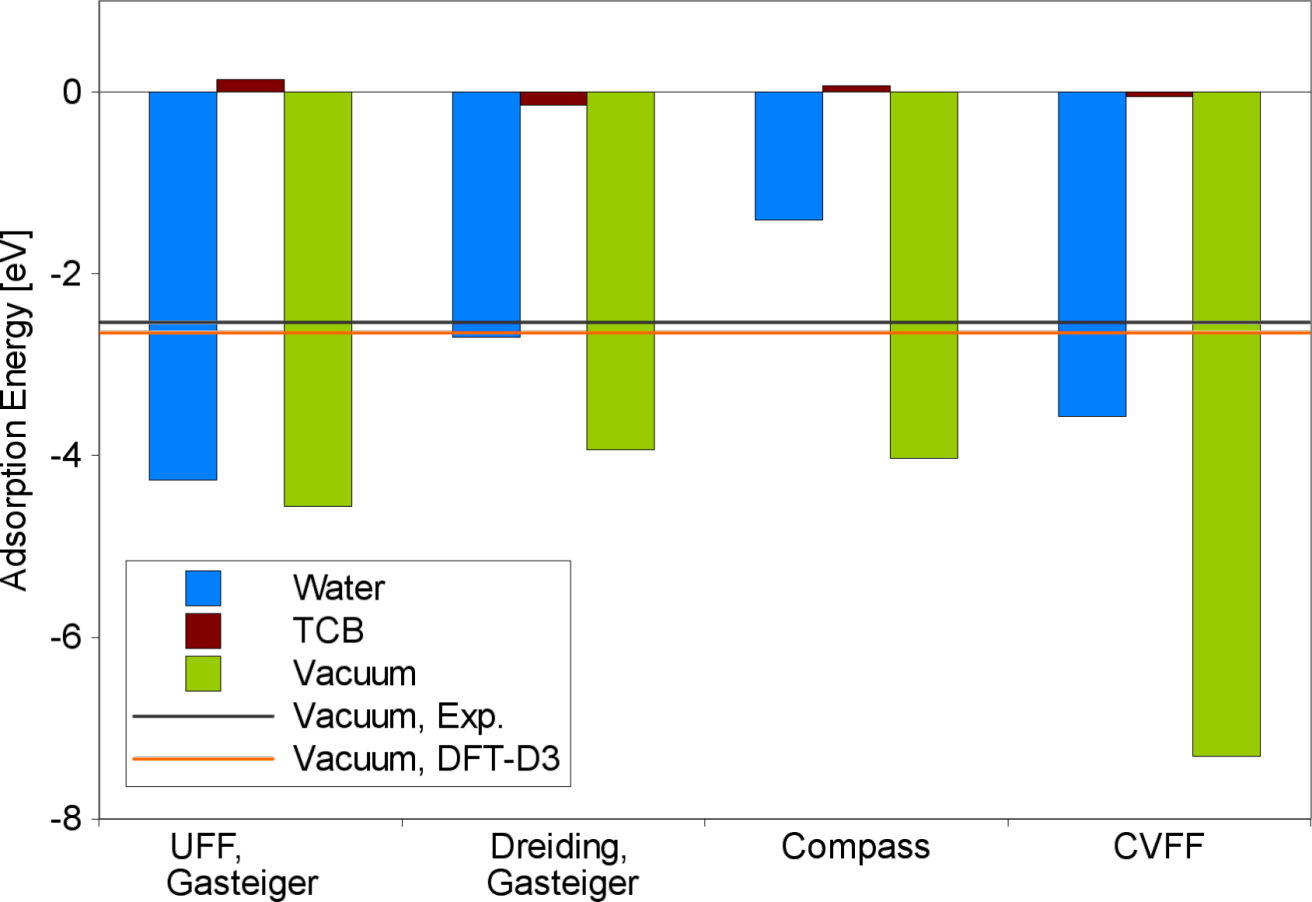
Adsorption energy of a 3,3′-BTP molecule on graphite: under vacuum conditions and at the solid/liquid interface from water or TCB, respectively. Experimental value and DFT-D3 result from [12].

Finally, we consider the adsorption energy of 3,3′-BTP on graphite under different conditions, namely for the BTP adsorption under vacuum conditions, at the solid/liquid interface with TCB as a solvent, as in the experiment, and additionally the case of adsorption of a BTP molecule from water. These numbers are listed in Table 1. Furthermore, in Figure 7 they are compared to the adsorption energy under vacuum conditions derived both from experiment and from DFT-D3 [36] calculations [12] with semi-empirical corrections for the van der Waals attraction. Obviously force fields significantly overestimate the interaction between graphite and the BTP molecule, as can be seen from the vacuum results [12]. Yet, as we will see below, for the adsorption from solution, results in semi-quantitative agreement with the experiment are still obtained.

The free enthalpies of adsorption were derived as illustrated in Figure 2: MD simulations were performed for a BTP molecule adsorbed on the surface with a solvent atmosphere at 298 K and for a dissolved molecule that is not yet adsorbed. The comparison of the average potential energies for the two different cases then yields the free enthalpy of adsorption of a dissolved molecule. The adsorption energies obtained following this procedure show surprising results: Even though the solvation and adsorption energies strongly vary with the force field, the general trend is the same in each case. While the adsorption from water leads to a high gain in energy, the adsorption from TCB is rather neutral in its energy balance. With UFF, adsorption from water yields over 90% of the energy that is obtained under vacuum conditions. Dreiding still reaches nearly 70%. With Compass and CVFF, this ratio drops to 35 and 49%, respectively. The adsorption energy from TCB is much smaller with all force fields, it ranges from 137 meV with UFF to −142 meV with Dreiding. This agrees qualitatively rather well with experimental findings, where the analysis of Langmuir adsorption isotherms has resulted in a 3,3′-BTP adsorption enthalpy of −340 meV at the solid/liquid interface [2]. In contrast to the observations under vacuum conditions, it might be that force fields tend to underestimate the interaction energy.

These findings can be rationalized fairly easily. BTP interacts strongly with the graphite surface via van der Waals interaction [12], thus the adsorption under vacuum conditions leads to a relatively high gain in energy. BTP also interacts strongly with the TCB solvent, which is why it can be dissolved in TCB. When it adsorbs on the surface, it gains the adsorption energy, but at the same time it loses part of the interaction with the solvent. In total, both contributions seem to balance out. The interaction between BTP and water, on the other hand, is rather weak. So in the hypothetical case of BTP adsorption from water, the system would gain a large adsorption energy, but the loss of BTP-water interaction is rather small, such that in total, an energy gain is associated with the adsorption.

### Phase stability

**Figure 8:**
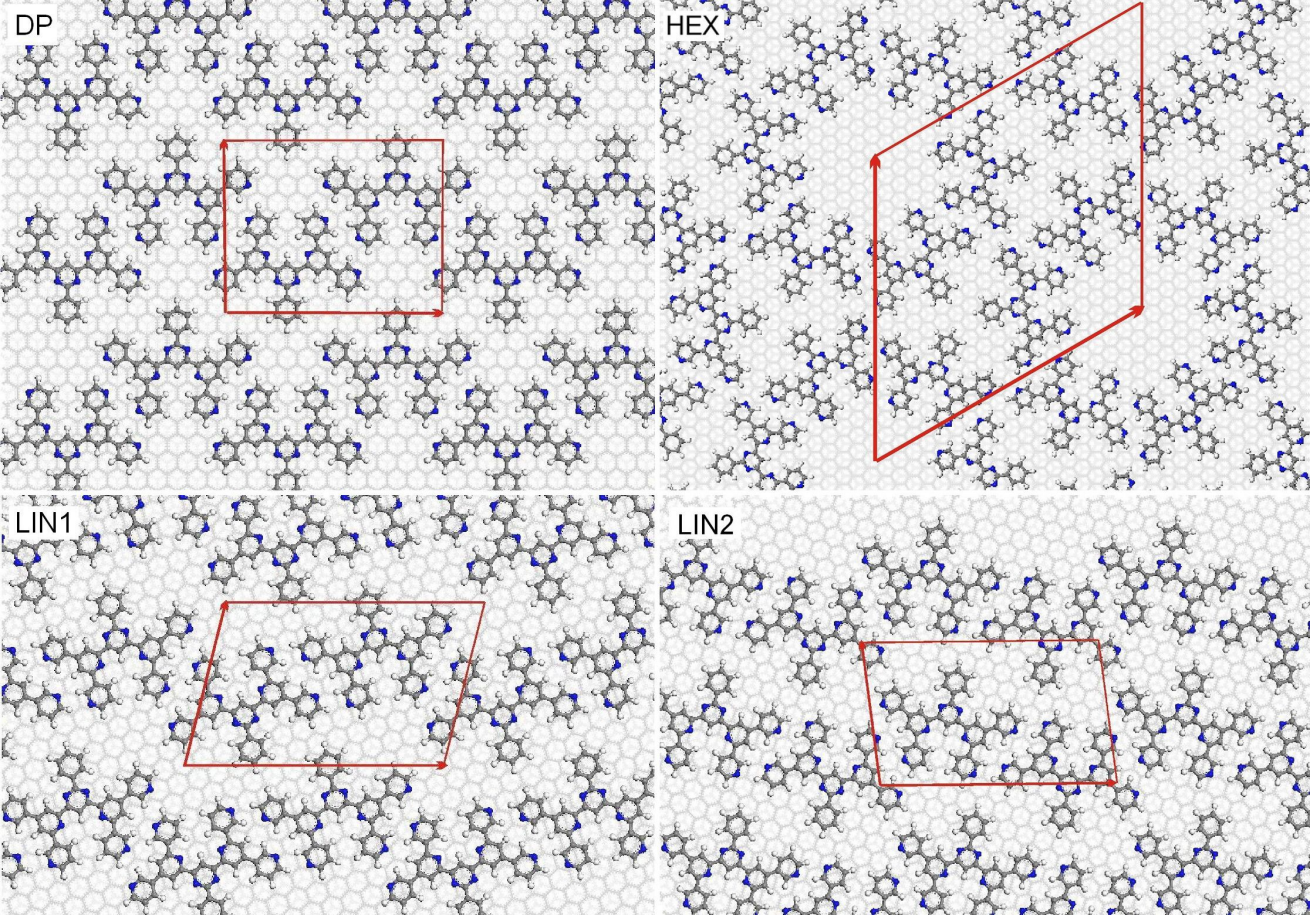
Structural models of the different 3,3′-BTP surface structures that have been observed at the liquid/solid interface.

Experimentally, it was observed that BTP on graphite in TCB solution exhibits a series of different structures, one hexagonal, two closely related linear, and one densely packed linear structure, that were ordered according to their packing density as a function of the concentration [6]. These structures are illustrated in Figure 8. Also the coexistence of different structures was found.

Thermodynamically, the stability of the adsorbate structures is governed by the free energy. Neglecting entropic effects, the free energy of adsorption can be expressed as [13,32]

[4]
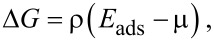


where *E*_ads_ is the adsorption energy per molecule in a given structure and ρ is the density of molecules per surface area in this structure. μ is the chemical potential, which depends monotonically on the concentration. A plot of the free energy of adsorption Δ*G* versus the chemical potential μ shows which phase is lowest in free energy at a given potential range. Using the experimentally derived adsorption enthalpy [2] of a single molecule and estimated values for the hydrogen bonding between the molecules, the sequence of observed structures as a function of concentration could be reproduced [2]. This sequence could also be reproduced based on adsorption energies obtained from force-field calculations at the gas/solid interface, but at an entirely different range of chemical potentials because of the rather different energy reference related to molecules in the gas phase.

The calculations presented so far have already shown that the inclusion of TCB into the model has a drastic effect. It may well also be that the solvent affects the strength of the intermolecular interactions. We have therefore estimated Δ*G* at 298 K taking the presence of the solvent into account.

MD runs with the full surface structures including graphite, BTP and TCB were carried out corresponding to an explicit solvation model. Due to the computational effort of these very large cells, some simplifications were necessary. Only three carbon layers could be used in order to represent the graphite surface. MD runs covered 150 ps, with the initial 50 ps as equilibration time. For each phase, one trajectory run for the adsorbed ordered surface structure in the presence of the solvent and another trajectory run for the BTP molecules in solution, as illustrated in Figure 2 using the same unit cell, were performed. Ideally, no interaction between a BTP molecule and the surface or another BTP molecule should occur in the latter simulations. These simulations were done by using the Compass force field, which provided the most reliable results.

As a result, the adsorption energies in Table 2 are obtained. These adsorption energies combine the BTP/graphite interaction with intermolecular interactions and solvent effects. The adsorption enthalpies range from 0.203 eV for the DP structure to −0.339 eV for the LIN2 structure. The Compass force field adsorption energy of a single BTP molecule amounts to 0.072 eV (see Table 1). This indicates that for the LIN1, LIN2 and HEX structures, whose adsorption enthalpies are negative, the intermolecular interaction is attractive, whereas for the DP structure the packing is so dense that the intermolecular interaction is already repulsive. The range of adsorption enthalpies is 80 meV larger than the range of adsorption energies of the corresponding structures at the gas/solid interface. Furthermore, at the gas/solid interface all structures are energetically more favorable per adsorbed molecule than the isolated adsorbed 3,3′-BTP molecule whose adsorption energy is −4.027 eV. This indicates that the intermolecular interaction is weakened by the presence of the solvent.

**Table 2:** Compass adsorption energy of 3,3′-BTP per molecule in different surface structures in electronvolts (eV) at the gas/solid interface and free enthalpy of adsorption at the liquid/solid interface at 298 K.

	DP	LIN1	LIN2	HEX

Vacuum conditions	−4.029	−4.173	−4.475	−4.491
Full solvation	0.203	−0.0775	−0.339	−0.0564

**Figure 9:**
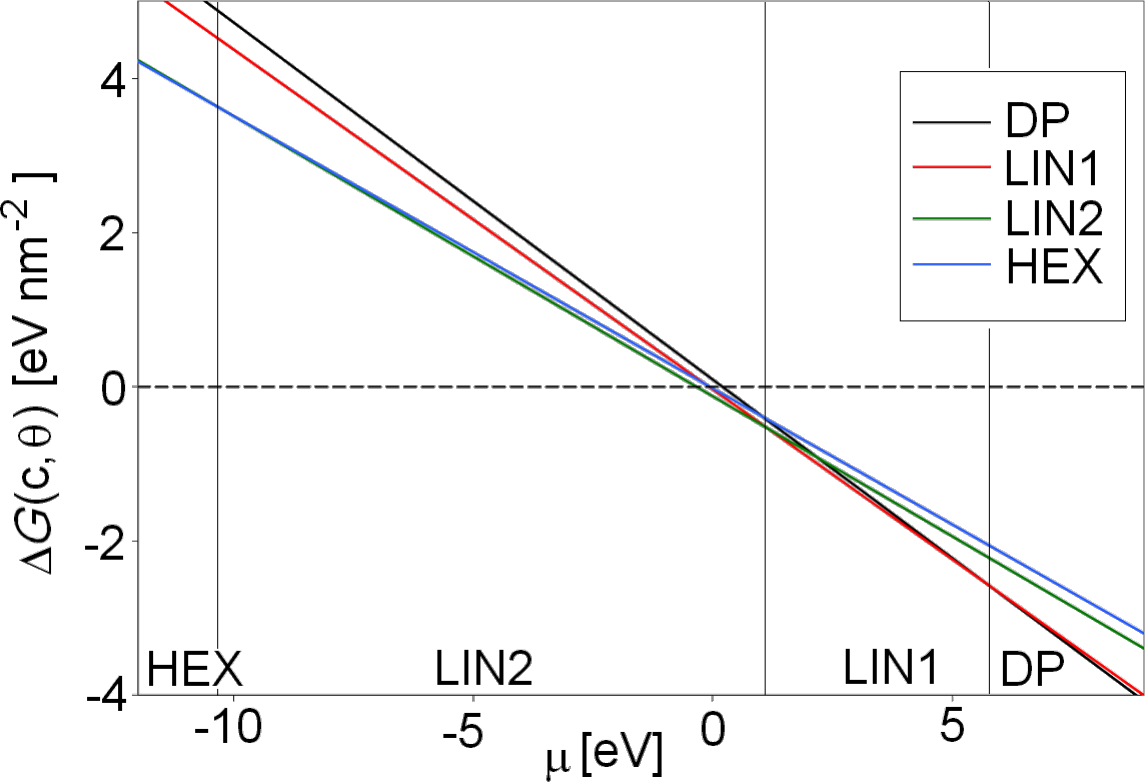
Plot of the free adsorption enthalpy of different 3,3′-BTP phases against the chemical potential obtained with fully solvated surface structures by using the Compass force field.

The phases in Figure 9 are ordered according to their packing densities, in agreement with the experiment. The broader range of adsorption energies now translates to a broad range of chemical potential values over which the phase transitions occur. In agreement with the semiempirical results, the transition between the LIN1 and LIN2 phases is found at a slightly positive chemical potential. However, compared to the semiempirical results, the HEX–LIN2 and LIN1–DP transitions occur at noticeably lower and higher chemical potentials, respectively. According to Figure 9, the HEX phase should not be observed since the free energy of adsorption is positive which means that the BTP-uncovered substrate is more stable. However, it is also apparent how close the two curves of the HEX and the LIN2 phases, on the one hand, and of the LIN1 and DP phases on the other hand are. Given the uncertainty of the force-field calculations, it may well be that the stability range of the LIN1 and LIN2 phases are smaller. It should also be noted that the stability ranges shown in Figure 9 are based on the assumption of thermal equilibrium. Kinetic effects in the structure formation are not taken into account, which may lead to the formation of metastable structures.

## Conclusion

The adsorption of 3,3′-BTP on graphite in the presence of water and TCB as solvents has been studied by molecular dynamics simulations at room temperature using various force fields. Whereas the results concerning water as a solvent show a wide spread between the different force fields, the results for TCB as a solvent are more consistent among the considered force fields. They all yield the result, in agreement with the experiment, that the adsorption of a single BTP molecule out of the TCB solvent is almost thermoneutral, i.e., it is not associated with a significant energy gain. Consequently, the formation of ordered hydrogen-bonded network structures of the BTP molecules in the presence of TCB as a solvent is mainly stabilized through the intermolecular interactions. The substrate basically only acts as a template allowing the planar arrangement of the BTP molecules. Finally, the stability of ordered BTP network structures on graphite at room temperature has been addressed within an atomic thermodynamics approach. In agreement with the experiment, four different phases are found to be ordered according to their packing densities as a function of the concentration of the BTP molecules in the solvent. However, the stability ranges of the linear phases seem to be too broad, caused probably by uncertainties in the force-field calculations.
